# atomium—a Python structure parser

**DOI:** 10.1093/bioinformatics/btaa072

**Published:** 2020-02-11

**Authors:** Sam M Ireland, Andrew C R Martin

**Affiliations:** Division of Biosciences, Institute of Structural and Molecular Biology, University College London, London WC1E 6BT, UK

## Abstract

**Summary:**

Structural biology relies on specific file formats to convey information about macromolecular structures. Traditionally this has been the PDB format, but increasingly newer formats, such as PDBML, mmCIF and MMTF are being used. Here we present atomium, a modern, lightweight, Python library for parsing, manipulating and saving PDB, mmCIF and MMTF file formats. In addition, we provide a web service, pdb2json, which uses atomium to give a consistent JSON representation to the entire Protein Data Bank.

**Availability and implementation:**

atomium is implemented in Python and its performance is equivalent to the existing library BioPython. However, it has significant advantages in features and API design. atomium is available from atomium.bioinf.org.uk and pdb2json can be accessed at pdb2json.bioinf.org.uk

**Supplementary information:**

Supplementary data are available at *Bioinformatics* online.

## 1 Introduction

Structural biology is the study of biological macromolecules at the molecular level, specifically the arrangement of their atoms in space, and how this atomic structure dictates their functions.

For any computational analysis of these structures, a representation of them must be stored on disk, and from the early days of structural biology, the PDB (Protein Data Bank) file format was used to represent these structures (Bernstein *et al.*, 1977). This format uses 80-character lines, with fields defined by position along that line, to represent information about the atoms in a structure. This includes information about the atoms themselves (their coordinates, names and connectivity), information about their organization (residue and chain information) and meta information about the structure such as how it was generated, who generated it and the experimental conditions.

In the case of the majority of structures, which are generated by X-ray crystallography, the coordinates of the atoms in these files represent the asymmetric unit—the repeating unit of the crystals. This may not be the biologically relevant structure, so these files contain biological assembly instructions: transformation matrices which are applied to the polymers in the structure to recreate the biologically relevant structure.

Over time, the limitations of the PDB file format have become apparent (Westbrook and Fitzgerald, 2005). Most seriously, the numeric atom identifiers are defined by a fixed-width field of five characters, meaning that the maximum atom ID is 99999, limiting the number of atoms a single file can contain. Initially this problem was not frequently encountered and, where it was, the structure was split over several files. Eventually, however, new file formats were introduced.

The mmCIF file format was introduced in 1997 as an extension to the existing Crystallographic Information File format. It uses a space-separated, linked table format to hold much more information than PDB files, and with no upper limit on structure size (Bourne *et al.*, 1997; Deshpande *et al.*, 2005). The PDBML format uses XML to represent structures (Westbrook *et al.*, 2005). Most recently, a binary form of mmCIF optimized for transmission over the web, MMTF, has also been introduced (Bradley *et al.*, 2017). The PDB file format has now formally been deprecated in favour of mmCIF, although it remains in widespread use.

Computational tools for processing these file formats and processing the models they represent are of great importance to structural biology. There are various examples for different languages, such as BioJava for Java (Lafita *et al.*, 2019) and BiopLib for C (Porter and Martin, 2015). These libraries provide the user with a standard interface for analysing very diverse structures, by representing them in terms of a small number of object types, such as atoms, chains and residues, and provide a layer of abstraction that makes more complex tasks such as creating scoring functions more straightforward. Python, a common programming language in Bioinformatics, has traditionally used the general-purpose library BioPython to parse these structure files (Cock *et al.*, 2009). However, there are limitations to this library, as will be outlined below.

Here we present atomium, a modern, lightweight, fast parser of .pdb, .cif and .mmtf files. It can read from, and save to, these file types and has powerful tools for processing and manipulating the structures they contain. It also makes PDB structures available in the JSON format using the pdb2json web tool, which is a wrapper around atomium. As JSON is a very widely used data representation format (particularly in transmission over the web), and as JSON parsing is part of the standard library of most programming languages, this additional tool makes the data contained in the PDB more easily accessible to those less familiar with the traditional file formats.

## 2 Materials and methods

### 2.1 atomium library structure

The inner workings of atomium can broadly be divided into two areas: the core structure classes for actually representing molecular structures, and the functions for parsing data from various file types and creating ‘models’ (i.e. data structures) from them.

**Fig. 1.**
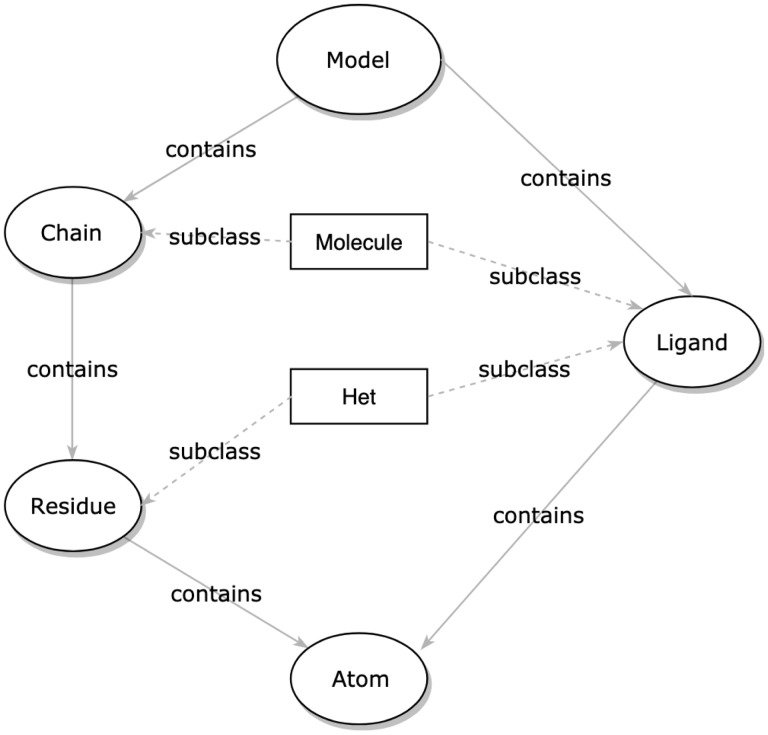
The relationship of structure classes in atomium, representing the hierarchy of types. While the structures can be created from scratch, this hierarchy has been designed to reflect the hierarchy of object types in PDB and mmCIF files

The core structure classes are how atomium represents molecular structures. These can in theory be used to create structures manually by creating each atom explicitly although, in practice, structure creation will usually be done by the parsers. There is a class for the top-level models themselves (the container that all the other structures inhabit) and for each of the sub-structure types usually encountered in PDB files. Consequently, there are classes for atoms, residues, ligands (non-polymer molecules) and chains (Fig. 1).

In every case where a structure has a collection of sub-structures within it (a chain’s residues, a residue’s atoms, etc.), these sub-structures are stored as a special atomium object called a StructureSet. These store the objects internally as a mapping of the structures’ identifiers (IDs) to lists of the structures so that lookup by ID can be done extremely quickly, but for all other purposes they behave as unordered sets. IDs are mapped to lists of structures rather than individual structures because it was necessary to allow duplicate IDs, usually found in biological assemblies.

### 2.2 atomium functionality

All structure classes can also use atomium’s filtering syntax. The objects can be filtered by any property, nested sub-property, by the regular expression of any string property (allowing for substring searches for example) or by numerical comparators of any numerical property (greater than, less than, etc.). For example, one can obtain all atoms of a given name, below or above a given charge threshold, or belonging to any residue of a given name or set of names (see Table 1).

**Table 1.** Examples of the filtering syntax that all atomium structures have by virtue of implementing the StructureClass metaclass

Command	Result
model.atoms()	All atoms
model.atoms(element=’N’)	All nitrogen atoms
model.atoms(mass__gt=14)	Atoms with mass greater than 14
model.atoms(name__regex=’CA—CB’)	CA and CB atoms
model.atoms(het__name__regex=’CYS—HIS’)	Atoms in cysteine and histidine residues
model.atoms(chain__length__lt=100)	Atoms in chains shorter than 100 residues

Among other operations, the atomic structures (a chain, a residue, a ligand, etc.) can all be transformed geometrically by translating or rotating; two atomic structures can be compared by measuring the RMSD between them, one can specify any atom or atomic structure and search for other atoms and atomic structures in the model which are, for example, within a given radius, or which have a particular property. For instance, the user can identify all sub-structures in a 5 Å radius of a given metal atom which are not water molecules, or identify all residues within 3 Å of a ligand that have a particular name. The documentation lists the full feature sets, and these are summarized in Table 2.

**Table 2:** Summary of the features that atomium sub-structures have, and the API for using them

Feature	API
Mass calculation	structure.mass
Relative elemental makeup	structure.formula
Centre of mass	structure.center_of_mass
Radius of gyration	structure.radius_of_gyration
RMSD	structure.rmsd_with(other)
Grid generation	structure.grid()
Atom proximity	structure.nearby_atoms(n)
Translation	structure.translate()
Rotation	structure.rotate
Water removal	structure.dehydrate()

As stated earlier, while the user is free to create these structures manually by accessing these classes directly, it is generally more convenient to create them by parsing structure files. atomium can read .pdb, .cif and .mmtf files. In each case, the overall process is the same:

1. Obtain the file contents as a string, either from the local filesystem, or remotely from the RCSB PDB servers (Rose *et al.*, 2010) (via HTTP) or a server (via SSH).
2. Determine which file type it is by looking at the file extension or, if not possible, by looking at file contents.
3. Convert the filestring to a Python dictionary whose structure is specific to that file type.
4. Convert that dictionary to a standard atomium data dictionary, whose structure is the same regardless of the file type origin.
5. Convert that data dictionary to an atomium File object with one or more models within it (NMR structures typically contain multiple models). Only one atom in a set of multiple occupancy atoms is used for the final model—currently the set with the alternate location identifier that comes first alphabetically (almost always A) is used, but future versions will allow this to be changed. Missing residue information is stored as a dictionary in the File object; this information comes from pdbx_unobs_or_zero_occ_residues rows and REMARK 465 records in mmCIF and PDB files, respectively.

Finally, atomium has the built-in ability to generate ‘biological assemblies’ from the coordinates given in PDB files. In the majority of structures, which are generated by X-ray crystallography, the coordinates of the atoms represent the asymmetric unit—the repeating unit of the crystals. This may not be the biologically relevant structure, so these files contain biological assembly instructions: transformation matrices which are applied to the polymers in the structure to recreate the biologically relevant structure. atomium can generate new models from the asymmetric unit coordinates using a single function.

### 2.3 pdb2json

**Fig. 2.**
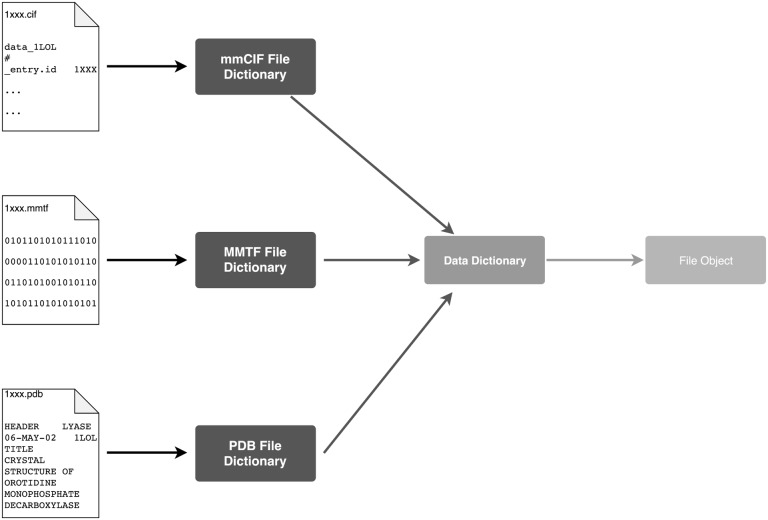
An overview of how parsing of the three file types is done. Because the three file types converge on a single data dictionary, all three file types can be represented as JSON

This process for parsing (summarized in Fig. 2) has a number of advantages over just trying to go from filestring to parsed object in one step. Making the three file types converge at one data structure (the atomium data dictionary) prevents duplication of effort involved in going from ‘data’ to ‘Python structure’. It also means that every file can have a consistent dictionary representation, which means that they can all be represented as JSON if desired. It is also easier for testing, as each stage in this (relatively complex) parsing process can more easily be tested in isolation.

As already noted, the process of parsing a structure file involves turning the raw filestring (or binary bytestring in the case of MMTF) into two successive Python dictionaries, before then being turned into a Python object. Initially this choice of Python dictionary as internal representation was a decision made to make development easier. However, Python dictionaries have a structure very similar to JSON, a data format that is frequently used for sending data over the web in a very human readable way, as it is essentially just nested key-value pairs. Thus, if the data dictionary is simply converted to JSON using Python’s built-in JSON library, atomium becomes a tool for turning any PDB structure into JSON.

pdb2json provides this facility. This is a simple, lightweight Django (v2.1, djangoproject.com) web app which uses atomium to take any PDB code and return the resultant structure as JSON. This is done using a URL, for example, /2SOD/ will return the JSON for the PDB 2SOD. It is currently available at pdb2json.bioinf.org.uk/.

The service is therefore an HTTP alternative to the FTP service mmJSON by PDBj (Bekker *et al.*, 2016), which provides FTP downloads of mmCIF structures only, and without the additional processing of the raw mmCIF table structures that pdb2json provides.

By default, pdb2json tells atomium to use the .cif representation for parsing, but this can be altered using, for example, /2SOD.pdb/. The structure of the JSON returned will be the same since atomium creates the same data dictionary regardless of file type, but some values may be different. For example, many .pdb files have titles, etc. in capitals whereas .cif files use title case, and atom IDs may be numbered slightly different.

If users so wish, they can obtain the initial file-type-specific Python dictionary as JSON by adding an argument called ‘file’ to the URL with no value, using (for example) the notation /2SOD/?file. This is generally of limited interest in the case of .pdb and .mmtf, except as a means of checking the original file contents pre-processing, but in the case of .cif, it can be very useful. This is because every attribute of the structure will be accessible in this dictionary, so if the subset of attributes atomium pulls out of files to annotate its final representation is not sufficient, other attributes can be obtained from this representation. For example, atomium File objects have the R-free and R-work attributes, but there are many metrics for these calculations in the original file, such as the number of reflections used to generate these numbers. pdb2json allows access to these metrics too.

PDB structures can be large and some are extremely large indeed. The user may not wish to download the JSON for an entire structure when they only need a single metric or set of metrics. Therefore, pdb2json allows the user to traverse the keys of the JSON structure using the supplied URL, if the user knows the identifiers for the relevant objects. For example, while /2SOD/ will return the JSON for the entire structure, /2SOD/quality/ will return only the quality sub-dictionary that was part of the original JSON object. This traversal can be as deep as the user wishes. For example, /2SOD/models/0/non-polymer/O.153/atoms/4382 will return information about a single zinc atom. In this case, this URL is the structure 2SOD, but only its first model (they are zero indexed), the non-polymer structure with ID O.153 in that model and the atom with ID 4382 in that non-polymer. This requires knowing the identifier of the atom and its containing HET record, but if these conditions are met, much smaller HTTP responses can be requested.

If JSON conversion is required offline, a pdb2json.py script is also provided in the atomium library itself. This is a simple utility which imports atomium, loads a file saved on disk, converts it to JSON and saves it.

## 3 Results

atomium is currently at version 1.0.3, the 22nd release. It is downloadable using the Python package manager PyPI and pip (pip3 install atomium), or by cloning the repository from GitHub directly (github.com/samirelanduk/atomium). The master branch always points to the most recent stable release, with new features being developed on separate branches.

**Fig. 3.**
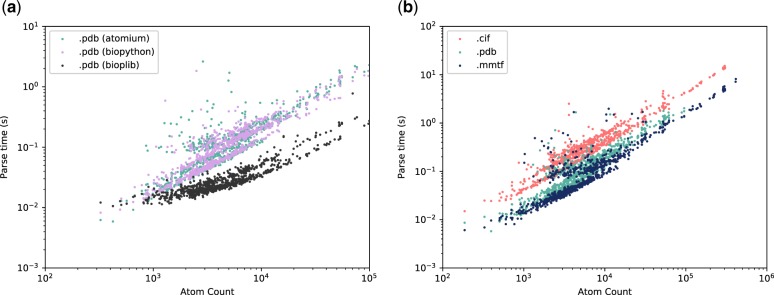
(**a**) A comparison of parsing speed between atomium, BioPython (both Python) and BiopLib (C) for the PDB file format. With occasional deviations, the two Python libraries are broadly equivalent. As expected, BiopLib is faster, particularly at higher atom counts, as it is written in a compiled language. (**b**) Time taken to parse the same 1000 randomly chosen single-model structures in the three file formats using atomium. Time as a function of atom count is linear and it can be seen that mmCIF structures take the longest, followed by PDB structures and MMTF structures

The speed of parsing (raw coordinates without assembly generation, as BioPython cannot do this) is comparable with BioPython for the .pdb file format. The two Python libraries were also compared with the C library BiopLib which, as might be expected, parsed the structures faster, particularly at higher atom counts (see Fig. 3a). The parsing time for the three file formats in atomium are of a similar order of magnitude, with .cif taking the longest (see Fig. 3b). In all five cases, the relationship between the number of atoms and the time taken to parse is linear and, for all comparisons, care was taken to ensure the same kinds of parsing were being done—no biological assembly generation, proper relationship parsing and assigning for the sub-structures, etc.

The SnakeViz profile visualization tool (SnakeViz v2.0.1, github.com/jiffyclub/snakeviz) can identify bottlenecks in parsing code, which has been useful in optimizing the atomium codebase. The increased time for .cif parsing can be partly explained using this tool as it identifies a bottleneck in scanning the lines of the file for embedded quotation marks. Because the file format allows for quite complicated nested quotation marks in lines, the algorithm used to handle them can be relatively time consuming and is a significant proportion of the overall parse time. The older PDB file format, for all its other deficiencies, has no such problem and nor does the newer binary MMTF format. Supplementary Figures S1–S3 show ‘profiles’ for each parse process, identifying which sub-functions take up most time in the overall process.

atomium has been used in the creation of the ZincBind database (Ireland and Martin, 2019), where its biological assembly processing capabilities were invaluable in identifying inter-chain zinc binding sites.

## 4 Discussion

Currently the general-purpose bioinformatics library, BioPython, is generally the structure parsing tool of choice for the Python programming language, but atomium offers three key advantages:

First, from a purely practical feature-set point of view, at the time of writing, BioPython does not have the ability to process the information contained in structure files’ biological assembly instructions, or create new models from them. This is a serious problem when dealing with structures whose asymmetric unit is markedly different from the biological assembly. For example, the insulin structure 1ZEH contains one subunit of the insulin hexamer, and the biological assembly instructions are required to make the true hexamer. BioPython cannot generate these structures by itself, which makes it unsuitable for examining interactions between chains. Atomium, however, can generate these with a single function, using the NumPy library optimized for matrix calculations. The structures generated from them will have duplicated IDs, but can still be selected individually by assigning novel names to them—particularly in the case of chain objects with no names assigned to them initially. While atomium does lack some features that BioPython offers, such as solvent accessible surface and residue depth, the future addition of such features is straightforward given the structural representation in atomium.

Second, and more philosophically, atomium adheres more closely to the Pythonic tenet that a piece of software should focus on doing one thing only and doing that one thing well. BioPython is a powerful, but general purpose, bioinformatics library with modules for many different bioinformatics applications. In contrast, atomium focuses solely on structural biology and specifically on the parsing, representation and saving of macromolecules. Its API, package structure, testing suite and documentation are all optimized around this purpose. On that basis, there is a strong argument that atomium itself should not be extended to include features such as solvent accessibility calculation since these are outside the remit of parsing and representing macromolecular structure.

Third, atomium has a simpler API than BioPython. There is no need to create a separate parser object; the whole parsing step can be done with the top-level functions atomium.open and atomium.fetch.

Finally, the addition of the pdb2json web server around atomium allows for access to the parsed contents of any PDB file through the browser in the widely accessible JSON format, removing the need for specialized parsers altogether if the user so wishes.

## 5 Conclusions

The atomium PDB parser is a novel, lightweight Python library which can handle three of the principal file types of structural biology, save changes made to them and generate the structures contained in their biological assembly instructions for more biologically realistic models. It contains powerful querying abilities for the models, as well as other useful metrics and tools.

## Funding

This work was supported by a Wellcome Trust PhD Studentship [203756/Z/16/A to S.M.I.].

*Conflict of Interest:* none declared.

## Supplementary Material

btaa072_Supplementary_Data
