# Supplementary material for: atomium—a Python structure parser
Source: Bioinformatics. 2020 Feb 11;36(9):2750–4. doi: 10.1093/bioinformatics/btaa072 (PMC7203745; doi:10.1093/bioinformatics/btaa072)
Supplement: btaa072_Supplementary_Data [file btaa072_supplementary_data.pdf]

# atomium — A Python structure parser
# Supplementary Material

Sam M. Ireland and Andrew C.R. Martin

December 11, 2019

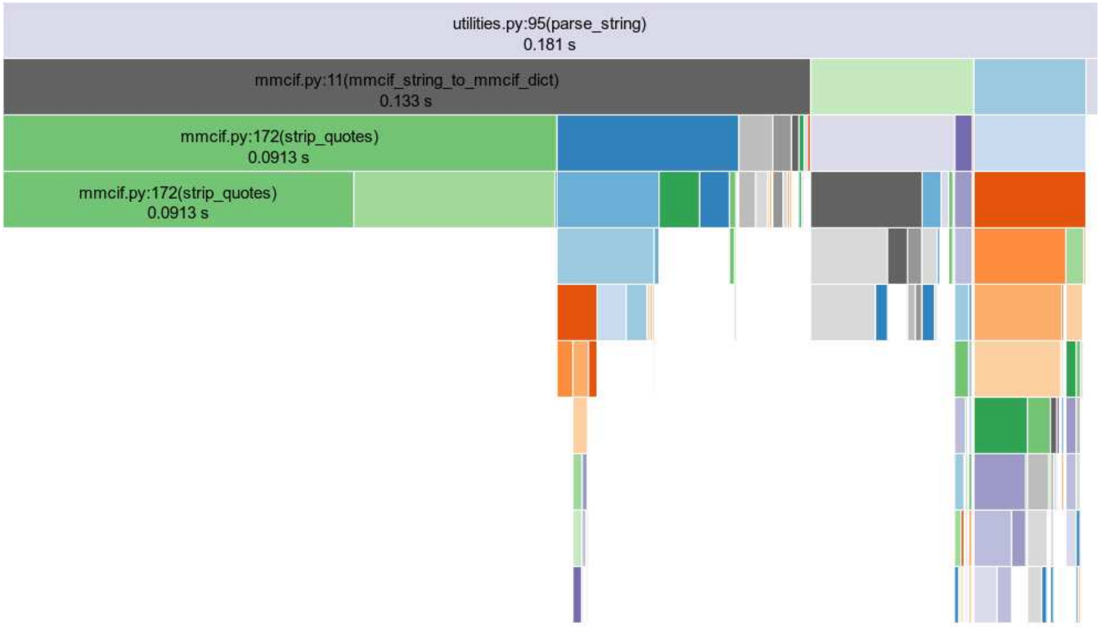


Figure 1: A visualisation of the 'profile' of mmCIF parsing. This shows how much time is taken by the different function calls in a single parsing event, which can be used to identify bottlenecks. The x-axis represents time — the more width a box takes up, the more time that function took to complete. If this is sufficiently large, the function name and the specific time taken are labelled. The y-axis represents the call stack — the top row is the parse function, the second row is functions called by the parse function, the third row is functions called by those in the second row, and so on, showing a tree-like structure of how each function allocates its time. The colours are solely to differentiate between the different functions more clearly. In the case of mmCIF, the `strip_quotes` function early on in processing takes up almost half the total parse time of 0.181 seconds.

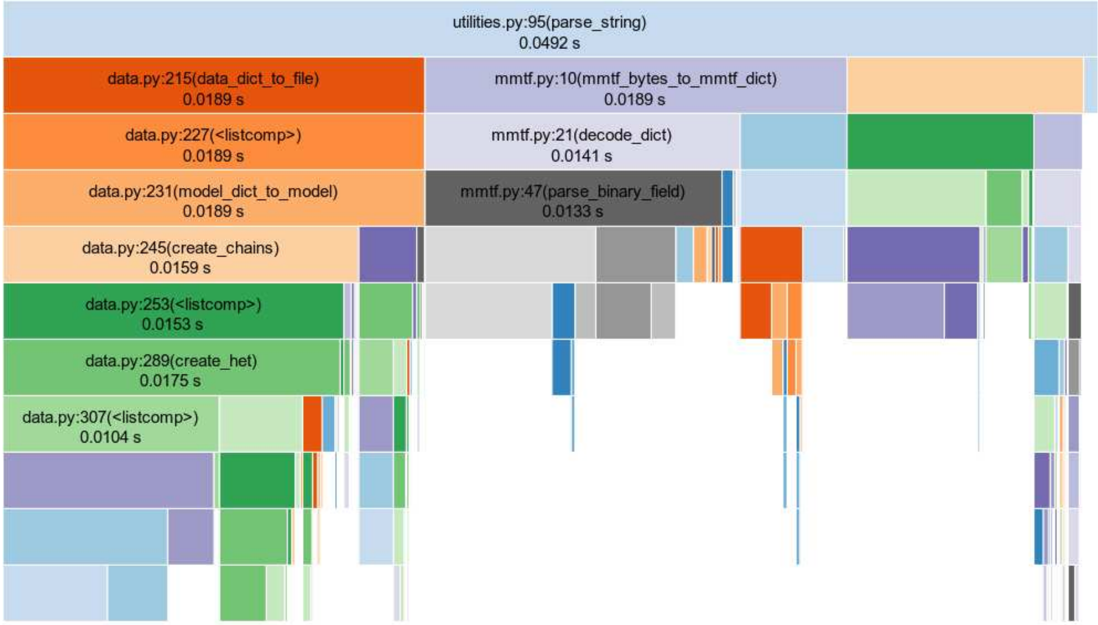


Figure 2: A visualisation of the profile of MMTF parsing. No single function is the principal bottleneck, and parsing is very fast.

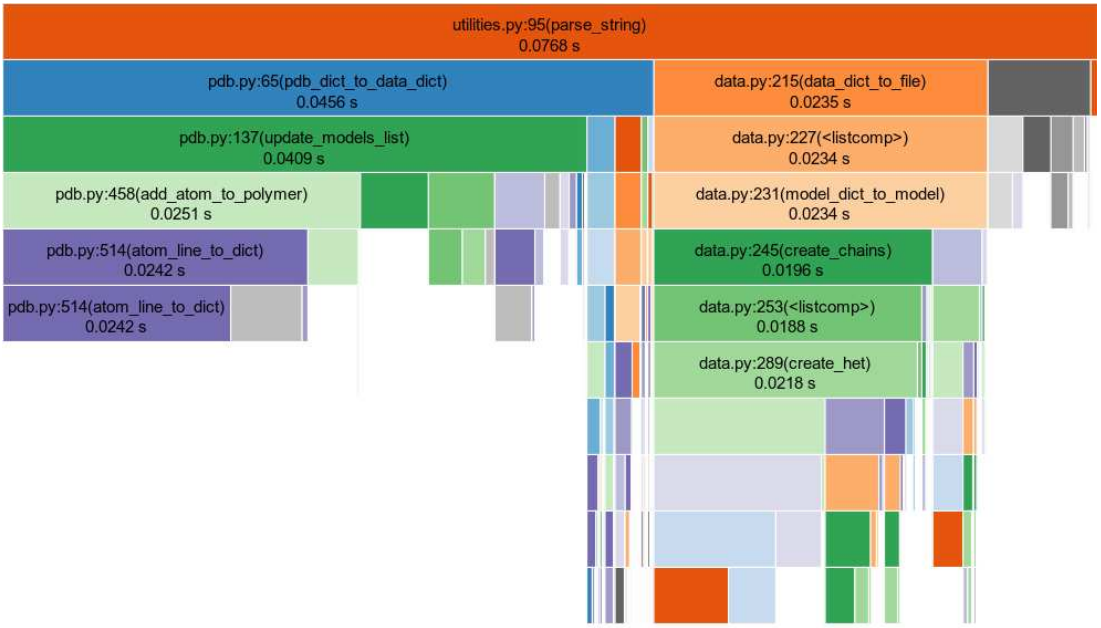


Figure 3: A visualisation of the profile of PDB parsing. Again there is no overall bottleneck.
